# A Microfluidic System for the Investigation of Tumor Cell Extravasation

**DOI:** 10.3390/bioengineering5020040

**Published:** 2018-05-23

**Authors:** Claudia Kühlbach, Sabrina da Luz, Frank Baganz, Volker C. Hass, Margareta M. Mueller

**Affiliations:** 1 Department of Mechanical und Medical Engineering, Hochschule Furtwangen University, Villingen-Schwenningen 78054, Germany; kuec@hs-furtwangen.de; 2 Department of Biochemical Engineering, University College London, London WC1E 6BT, UK; f.baganz@ucl.ac.uk; 3 Hahn-Schickard, Villingen-Schwenningen 78054, Germany, sabrina_daluz@yahoo.de; 4 HFU Hochschule Furtwangen, Department Medical and Life Science, Villingen-Schwenningen 78054, Germany

**Keywords:** microfluidic device, HPAEC, tumor cell extravasation

## Abstract

Metastatic dissemination of cancer cells is a very complex process. It includes the intravasation of cells into the metastatic pathways, their passive distribution within the blood or lymph flow, and their extravasation into the surrounding tissue. Crucial steps during extravasation are the adhesion of the tumor cells to the endothelium and their transendothelial migration. However, the molecular mechanisms that are underlying this process are still not fully understood. Novel three dimensional (3D) models for research on the metastatic cascade include the use of microfluidic devices. Different from two dimensional (2D) models, these devices take cell–cell, structural, and mechanical interactions into account. Here we introduce a new microfluidic device in order to study tumor extravasation. The device consists of three different parts, containing two microfluidic channels and a porous membrane sandwiched in between them. A smaller channel together with the membrane represents the vessel equivalent and is seeded separately with primary endothelial cells (EC) that are isolated from the lung artery. The second channel acts as reservoir to collect the migrated tumor cells. In contrast to many other systems, this device does not need an additional coating to allow EC growth, as the primary EC that is used produces their own basement membrane. VE-Cadherin, an endothelial adherence junction protein, was expressed in regular localization, which indicates a tight barrier function and cell–cell connections of the endothelium. The EC in the device showed in vivo-like behavior under flow conditions. The GFP-transfected tumor cells that were introduced were of epithelial or mesenchymal origin and could be observed by live cell imaging, which indicates tightly adherent tumor cells to the endothelial lining under different flow conditions. These results suggest that the new device can be used for research on molecular requirements, conditions, and mechanism of extravasation and its inhibition.

## 1. Introduction

One of the characteristics of malignant cancer is that it can form metastasis in distant organs by tumor cell invasion and the destruction of surrounding tissue [1].

This process is characterized by three indispensable, very complex actions, namely: (i) the dedifferentiation of tumor cells allowing their migration into the metastatic pathways, that is, the circulation [2,3,4,5,6]; (ii) their passive distribution into distant organ systems; and (iii) the transendothelial migration into the surrounding tissue to expand to secondary metastatic tumors [2,3,4,5,6]. The mechanism of extravasation is not yet fully understood, but is thought to resemble the recruitment of leukocytes during an inflammatory response. Critical steps in both processes are the rolling of tumor cells on the inner vessel lining, the tight adhesion to the endothelial cells, and the transendothelial migration [7,8].

Classical cell culture models, while easy to use, do not incorporate the important aspect of cell- and matrix-interactions in a three dimensional (3D) tissue context [9,10,11]. The 3D cell culture models, which incorporate cell–cell and cell–matrix interactions, and organotypic structures, which more closely resemble the in vivo situation, address this problem [9,10,11]. A novel approach for 3D cell culture models is the adoption of microfluidic systems, which allow highly reproducible experiments in small volumes of liquids that can be easily controlled [12,13,14].

### 1.1. Cancer Metastasis

During the process of metastasis, the intravasation initiates with the increased motility of primary tumor cells that migrate from the primary tumor site to the blood or lymphatic circulatory system [15,16]. When tumor cells reach the vessel, they intravasate a process that requires an active translocation of tumor cells through the barrier of the extracellular matrix and the endothelial lining [15,16]. In the vessel system, the tumor cells are distributed passively, until they reach the metastatic site in the distant organ system, where they extravasate again. This process requires their interaction with surface receptors of the endothelium, which results in a signal transduction that initiates the extravasation process into the surrounding tissue where the tumor cells then create secondary tumors [3,7,15,16,17]. Only about 1% of the migrating tumor cells establish a distant metastasis [3,7,17]. It is assumed that this process is regulated by the activation and deactivation of several specific genes, including the so called metastasis-suppressor genes, that regulate the development of metastasis but do not influence the tumor growth at the primary site [16,18]. A detailed analysis of the extravasation process reveals three distinct steps, namely: (i) the rolling of cancer cells on the endothelium that activates the endothelial cells, (ii) their tight adhesion to the vessel wall, and (iii) the transmigration through the endothelial monolayer [7,8].

Two different models describe the mechanisms that regulate the adhesion to the vessel wall and extravasation. The ‘seed and soil’ hypothesis, proposed by Stephen Paget in 1889 [19], claims that the homing of metastatic cells (i.e., seed) requires the interaction with the microenvironment of their target organ (i.e., soil) [15]. Another hypothesis claims that the extravasation entrapment of circulating tumor cells in small capillaries is sufficient [17].

For both models, intimate contact between the tumor cells and endothelial cells is essential to allow adhesion to the vessel wall and subsequent transendothelial migration (TEM). While some aspects of tumor cell extravasation resemble the leukocyte TEM during inflammation, the exact mechanism of contact, adhesion, and TEM of tumor cells are not yet fully understood [7,8]. It becomes abundantly clear that the chemokines and their receptors play a crucial role in every single step of the metastatic cascade [8,16,20], an aspect that can easily be studied in microfluidic devices by the selective addition and blockade of these pathways. 

### 1.2. Microfluidic Devices

Two dimensional (2D) tissue culture models, even when coated with extracellular matrix proteins, are of limited use in mimicking the in vivo conditions [21,22,23], as they lack any structural or mechanical parameters. In contrast, 3D models that incorporate cells into an extracellular protein matrix, allow the interaction between the tumor cells and their microenvironment [22,23]. One of the most popular static 3D approaches is the Boyden Chamber Assay, where two medium-filled compartments are separated by a porous (sometimes protein coated) membrane that allows the cells to migrate from the upper compartment, through the membrane’s pores, into the lower compartment. The number of cells and the time that is needed to reach the lower compartment can be correlated to the malignancy of the cancer cells [24].

However, this model is still not suitable for the investigation of the dynamic effects of cell–cell interactions, spatial organization, and cell migration [22,23], and cannot provide any insights in the complexity of the multistep process of cancer metastasis [21]. To better understand this stepwise process, more sophisticated models are much in need. Microfluidic devices combine the advantage of a 3D-model with dynamic flow conditions, as found in vivo, while allowing standardized, highly reproducible experimental conditions that can provide a basis for high throughput screenings [12,13,14]. As such, they are well suited for research on the metastatic cascade [25], including the interaction of tumor cells with the endothelial cells of the vessel wall and the influence of forces within the blood stream [21]. In this context, several studies on TEM, using microfluidic models, were published within the last ten years, which suggest an increase in TEM by the application of flow to the tumor cells [26].

One frequently used approach that creates a microvascular network within the microfluidic channel, is taking advantage of the capability of the endothelial cells to create self-assembled tubule-like structures [27,28,29]. These types of microfluidic models often embed the vascular cells in a matrix of extracellular proteins, like Collagen I, laminin, or fibrinogen [17,30,31,32]. While the tubule-like structures resemble the capillaries in vivo, they can usually not be subjected to flow and can thus only allow the study of the tumor cell extravasation under static conditions [17,29].

However, there are approaches that allow the application of varying flow rates in an endothelial cell lined vascular equivalent. One possibility is to introduce the endothelial cells as a monolayer into a microfluidic channel, which is often coated with matrix protein-like poly-D-lysine [17,31] or matrigel [33,34], for better adhesion properties. Independent of the model geometry or of the type of endothelial cells that are used, most of these devices are made of Polydimethylsiloxane (PDMS), which is frequently activated by plasma-treatment and is bonded to glass [17,31,33,34]. For example, Zervantonakis et al., 2012, describe a model consisting of two parallel channels that are separated by a hydrogel matrix that contains human endothelial cells from an umbilical cord vein (HUVEC) and fibrosarcoma and breast cancer cells. In this model, the tumor cells migrate towards the endothelial cells and intravasate without adding any kind of flow [35]. A similar model was proposed by Haessler et al., 2012. Here, two adjoining channels were coated with poly-D-lysine and were subsequently filled with hydrogels of bovine collagen type I, either with or without embedded breast cancer cells. Hydrogels of different permeability were used to control the interstitial flow [26], which was shown to influence the migration behavior and migration speed of the breast cancer cells [36].

Another approach to study the adhesion of the tumor cells to an endothelial monolayer was published by Song et al., 2009. The device consists of two PDMS layers with a porous membrane sandwiched in between. The membrane with a pore size of 400 nm prevents the transmigration of the tumor cells, but allows for the diffusion of soluble factors. The upper channel was seeded with human dermal microvascular endothelial cells (HDMVEC), from foreskin and human breast cancer cells, which were introduced into this channel via an inlet. Chemokines could be added to the lower channel under different flow conditions. The expression of CXCL 12 and its corresponding receptor CXCR 4 by the tumor cells was shown to promote tumor metastasis, potentially by a CXCL 12 induced upregulation and activation of adhesion molecules in endothelial cells, which supports the interactions with the circulating tumor cells [36]. Accordingly, in this study, tumor cells preferentially adhere to endothelial cells treated with the chemokine [36].

Using a similar device made from PDMS, Zhang et al., 2012 studied the transmigration of the tumor cells through an endothelial layer, into a second channel that was coated with basement membrane proteins under static conditions [37]. In this microfluidic device, small aggregates of a salivary gland adenoid cystic carcinoma cell line have required the addition of CXCL12 chemokine as a ‘homing factor’ for successful transmigration [37].

Jeon et al., 2013 presented a similar model with two channels separated by a gel region containing type I collagen. One channel is coated with poly-d-lysine and seeded with HDMVEC and the second channel serves as medium channel. The extravasation of breast cancer cells from the first channel into the gel region of the second one could be observed after one day under static conditions [17].

While these studies were mostly done under static conditions, there are a have been reports describing the addition of flow to microfluidic systems with endothelial cells. In the study by Shin et al., 2011, every second day, 10 mL of buffer was infused at a flow rate of 88 µL/min to the channel [33], whereas Riahi et al., 2014 added tumor cells, at a flow rate of 50 µL/h, to the HUVEC endothelial cell lined suspension flow channel. After adding the tumor cells, the flow rate was lowered to 1 µL/h to allow the adhesion to the endothelial cells. In this study, the tumor cells transmigrated from the suspension flow channel through the endothelial monolayer into a channel containing chemokines in matrigel [34].

Just recently, Cui et al. published a microfluidic device consisting of two independent flow channels with a porous membrane that was sandwiched in between with several cell collection chambers underneath an endothelial monolayer. The membrane, with pore sizes ranging from 10 to 26 µm, was coated with matrix proteins for better adhesion properties and was seeded with primary endothelial cells from the foreskin (HDMVEC). The tumor cells—a human breast cancer cell line—were injected to the overhead chamber and a flow of 20 µL/min of culture medium was added. CXCL12 chemokine was added to the channel underneath the endothelial cell monolayer, which acted as chemo attractant to the tumor cells. After an incubation time of 15 h, the migrated tumor cells were collected and counted. While the system principally seems well suited to study the tumor cell extravasation, the authors describe the confluency of the endothelial cells lining the channel as a major problem. Only the single subareas, where the endothelial cells showed a total confluency on top of the membrane could be used for the analysis of the transmigration of tumor cells [38].

### 1.3. Aim of the Study

In this study we introduce a new microfluidic device for the analysis of the different processes during the extravasation of tumor cells from blood vessels, and the interaction of the tumor cells with the endothelial lining of blood vessels under dynamic conditions.

**Figure 1:**
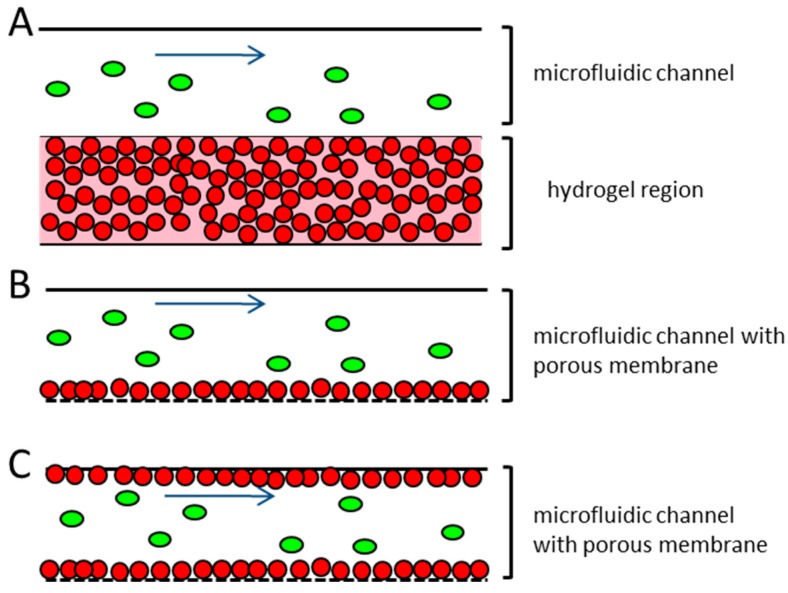
Schematic representation of different microfluidic systems used for the research on the vessel associated steps of the metastatic cascade (**A**) endothelial cells are embedded in hydrogel and injected into the microfluidic channel, where they are arranged in a capillary network. Tumor cells are introduced to the top of the hydrogel, and can migrate into it. No perfusion of the tubule-like structures takes place; (**B**) Endothelial cells are seeded in a monolayer on top of a porous membrane. The confluency of the endothelial cells on the membrane can often not be guaranteed. The tumor cells are introduced to the system under flow conditions; (**C**) Proposed microfluidic device. Endothelial cells are seeded to the total dimension of the microfluidic channel and the adjacent porous membrane, representing the vessel equivalent. The confluency of the endothelial cells is confirmed before the experiment. Tumor cells are introduced to the device under different flow conditions. Red dots—endothelial cells; green ovals—tumor cells; blue arrow—direction of flow.

Several published microfluidic approaches to address this problem use endothelial cells that have been embedded in hydrogel and take advantage of their ability to self-assemble into tubule-like structures [27,28,29]. A disadvantage of these systems is the multilayered structure that frequently occurs when the endothelial cells are embedded into a hydrogel (Figure 1A). This results in a non-physiological barrier that the extravasating tumor cells would have to pass through. This does not correspond to the in vivo situation, where the blood or lymphatic vessels contain only a monolayer of endothelial cells [39]. Other microfluidic devices working with endothelial monolayers seed the endothelial cells on top of an extracellular matrix protein coating, either on a porous membrane or on a hydrogel (Figure 1B). The devices usually consist of closed channels, where the endothelial cells’ suspension are injected and incubated, until they adhere to the device material. In these devices the confluency of the endothelial cells within the microfluidic channel are often patchy and hard to control, thereby creating some difficulties, since the whole dimension of the microfluidic channel cannot be used for the extravasation experiments [17,31,36,38].

The proposed microfluidic device aims to improve these issues. The device is intended for the investigation of all of the steps of the extravasation process, including the rolling of the tumor cells on the endothelial cells, tight adhesion to the endothelial lining, and transendothelial migration. The microfluidic device will be seeded in a monolayer with primary endothelial cells from the target organ of the metastatic tumor cells used, for example, lung. The endothelial cell confluency can be easily monitored along the whole length and on all of the sides of the microfluidic channel, in order to achieve optimal cell–cell contacts of the endothelial cells and introduced tumor cells. The perfusion of the tumor cells into the endothelial cell lined vessel equivalent can be done under different flow conditions.

We will demonstrate the establishment of a stable endothelial monolayer in the device, without the addition of any matrix proteins, and report the initial experiments of characterizing the tumor cell adhesion under flow conditions.

## 2. Materials and Methods

### 2.1. Cells, Cell Lines, and Cell Culture

**Figure A1:**
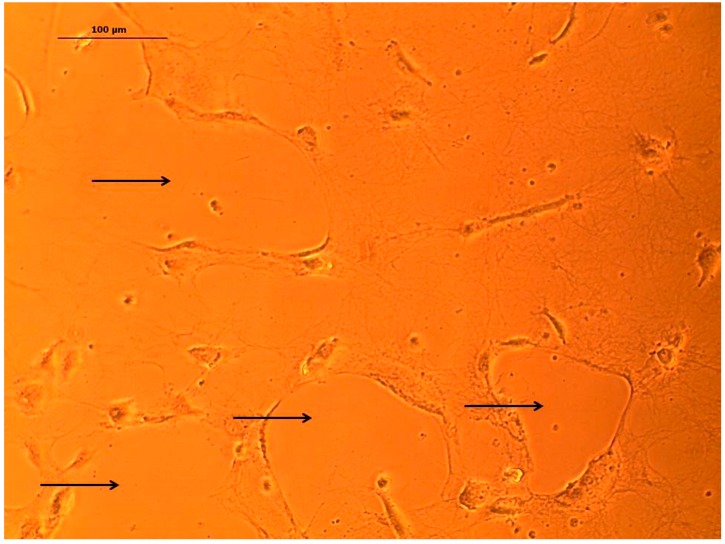
Example image of HPAEC endothelial cells disintegrating collagen coating after incubation time of 24 h. Within the culture, holes within the collagen layer can be observed (black arrows). Scale bar 100 µm.

Human primary pulmonary arterial endothelial cells (HPAEC; Figure A1), green fluorescent protein (GFP) expressing lung carcinoma cells H838 (H838GFP), and SK-Mel 28 malign melanoma cells (SK-Mel 28GFP) were used for this study. The HPAEC were isolated from the pulmonary artery tissue (Pelobiotech, Planegg, Germany). The HPAEC were cultured in T75 culture flasks, with Microvascular Endothelial Cell Medium (Pelobiotech, Germany) and were subcultivated when there was >80% confluent. In order to loosen the cell–cell connections. 0.05% Ethylendiamintetraacetate (EDTA) was used and 0.1% Trypsin/EDTA mix (1:1) was to singularize the cells. The seeding concentration was 5–10 × 10^3^ cells per cm^2^ growth area. The tumor cells that were used for this study were H838 non-small cell lung cancer cells and SK-Mel 28 melanoma cells. H838 as well as SK-Mel 28 tumor cells were stably transfected with the plasmid pTracerTM-CMV2 vector (Life Technologies, Darmstadt, Germany) in order to express the GFP and the clones that were characterized to exhibit similar characteristics (proliferation, migration, Boyden Chamber Invasion, 3D organization) to the parental cell line were used for further experiments. The GFP transfected lung carcinoma cell line H838GFP and the GFP transfected melanoma cell line SK-Mel 28GFP cells were cultured in 10 cm culture plates with Dulbecco’s Modified Eagle Medium (DMEM) culture medium, which contained 10% fetal calve serum (FCS), 1% penicillin/streptomycin, and 200 µg/mL zeocin as selective antibiotics, at standard conditions (37 °C, 5% CO_2_). H838GFP cells were subcultivated by incubation with 10 mL of 0.05% EDTA for 5 min, which was followed by singularizing the cells using 3 mL of 0.025% Trypsin/EDTA mix (1:1) for 3 min. The SK-Mel 28GFP cells were also incubated with 0.05% EDTA for 3 min and singularized, using 0.01% Trypsin/EDTA mix (1:1) for 1 min. The reaction was stopped with a 7 mL culture medium. The seeding concentration of the tumor cells was 3 × 10^6^ cells in a 10 cm culture plate, containing 10 mL of medium.

### 2.2. Fabrication and Testing of the Microfluidic Device

#### 2.2.1. Identification of Best Suitable Materials

For use in the microfluidic device, different plastic materials were tested for biocompatibility, optical transparency and quality, mechanical processing possibilities, commercial availability, and the potential for steam sterilization. For the experiments, the materials were autoclaved before seeding with HPAEC. Before the seeding process, some of the materials were coated with FCS, collagen, or laminin, so as to improve the cell adherence. HPAEC cells were seeded in a concentration of 7000 to 10,000 cells per cm^2^ and were cultivated under standard conditions for a period of at least four days. The results were evaluated photographically and the semiquantitative values (0%, 50% and 100%) for the confluency rates that were achieved after three to four days were allocated.

#### 2.2.2. Device Fabrication

**Figure 2:**
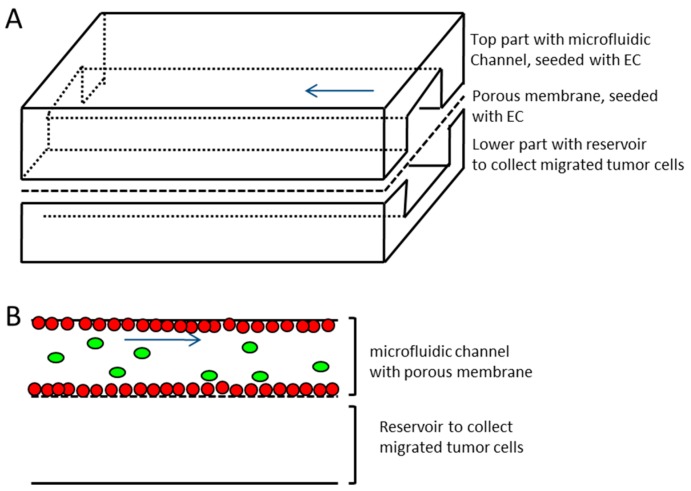
Schematic representation of the microfluidic device. (**A**) Upper and lower channel made of Polydimethylsiloxane (PDMS) with porous membrane (pore size 5 µm) sandwiched in between. The upper channel with membrane represents the vessel equivalent (channel dimensions 500 µm × 100 µm × 5.9 cm, width × height × length, respectively) and is seeded with endothelial cells (EC), the lower channel represents reservoir for transmigrating tumor cells (lower channel dimensions 1 mm × 1 mm × 5.9 cm, width x height × length, respectively). (**B**) Side view sketch of microfluidic channels and membrane separating the two channels. Red dots—endothelial cells growing on all sides of the upper channel and on the membrane; green ovals—tumor cells introduced to vessel equivalent; blue arrow—direction of flow.

The microfluidic device consisted of three parts (see Figure 2), namely, an upper channel which represented the vessel equivalent, a lower channel to collect the extravasated tumor cells, and a porous membrane (it4ip SA, Louvain-la-Neuve, Belgium), which separated both of the channels. The membrane was made from Polyethylene terephthalate (PET), had a thickness of 19 µm, a pore size of 5 µm, and a pore density of 6 × 10^4^ cm^−2^. The slides that contained the channels were fabricated by replicate molding of PDMS (Dow Corning, Midland, MI, USA). The mold was manufactured by the Institute of Micro- and Information-Technology of the Hahn–Schickard–Gesellschaft e.V., Germany. After the curing process, the device was oxygen plasma treated and autoclaved. The upper channel had a dimension of 500 µm × 100 µm × 5.9 cm. The endothelial cells were seeded separately on the single parts, which were assembled just before the experiment. The three parts were tightly clamped in an aluminum frame, which made additional sealing (e.g., by bonding of the device) unnecessary. A low pressure syringe pump (Cetoni, Korbußen, Germany) was connected to the upper channel inlet, which allowed continuous or pulsatile flow rates for medium or tumor cell suspension, which ranged from 0.4 to 1.2 µL/s (flow velocities ranging from 8 mm/s to 24 mm/s).

#### 2.2.3. Cell Seeding

The HPAEC were singularized, as described above, and seeded with 1 × 10^6^ cells to the upper channel and the top of the porous membrane, and were incubated for 24 h at standard conditions.

### 2.3. Tumor Cell Suspension for Perfusion Experiments

For the tumor cell suspension that was perfused into the microfluidic channel, H838GFP or SK-Mel 28GFP cells were subcultured in the DMEM culture medium, which contained 10% FCS and 1% penicillin/streptomycin. The tumor cell concentration for the perfusion of the microfluidic device amounted to 0.5 × 10^6^ cells per mL.

### 2.4. Flow Experiments

The assembled microfluidic system was connected to the low pressure syringe pump. The maximum flow velocity for the medium flow or influx of the tumor cell suspension ranged from 8, 12, 16, to 24 mm/s, either with a continuous or pulsation frequency of 60/min. The pulse duration was 500 ms, which was followed by a stop of flow for 500 ms. The duration of the experiment ranged from 6 to 72 h, as stated in the results section.

After finishing the experiment, the microfluidic device was disassembled for further analysis, such as immune fluorescence staining or counting of adherent tumor cells in different areas over the whole length of the microfluidic channel, which omitted the inlet and the outlet. For the disassembly, the clamp that held the aluminum frame was taken off and the microfluidic device and the three single parts were taken apart.

### 2.5. Immunofluorescence

Before the staining, the probes were incubated in 4% paraformaldeyde (PFA, in 1× phosphate buffered saline (PBS)) for 20 min, which was followed by the treatment with 70% and 100% ethanol for 5 min each. The blocking of the probes was executed with 18% bovine serum albumin (BSA) for 1 h. The primary antibodies were incubated overnight at 4 °C. For the negative control, just 18% BSA without the primary antibody was added to the probe. The negative control was stained on the same probe, either slides, or the material of the microfluidic device. The experimental conditions were the same for the negative and the positive group. The next day, three washing steps with 1× PBS for 5 min, to remove the unbound primary antibodies, were carried out. The secondary antibodies were added and incubated for 1 h at room temperature. After three more washing steps, the nuclei were stained with Hoechst dye (Sigma-Aldrich, Albuch, Germany) and were diluted 1:1000 with 1× PBS for 5 min. This was followed by three more washing steps with 1× PBS, which was followed by embedding the probes with Dako Fluorescent Mounting Medium (Agilent, Waldbronn, Germany). The staining was microscopically evaluated. Primary antibodies used were Anti-VE-Cadherin in a concentration of 1:200 in 18% BSA (from rabbit, Sigma-Aldrich, Germany) for the detection of adherence junctions, which were the cell–cell connections in the endothelial cells [40] and Anti-Collagen IV in a concentration of 1:50 in 18% BSA (from rabbit, Progen, Heidelberg, Germany) for the detection of the extracellular matrix protein collagen IV. This was the main component of the human basal membrane [41,42]. The secondary antibody was used in a concentration of 1:500 in 18% BSA (Cy 3 donkey, anti-rabbit, Dianova, Hamburg, Germany).

### 2.6. Data Analysis

The number of adherent tumor cells to the endothelial lining was determined by counting the adherent tumor cells at the top of the channel and on the membrane on a length of 2 mm at five different positions, each. The mean values were taken from the 10 single counts per microfluidic device and the results were extrapolated to mean values/mm^2^. The standard deviation was taken over the different devices that were used in the experiment. Either two or three replicate experiments were performed, as stated in the results section.

## 3. Results

### 3.1. Design and Fabrication of the Microfluidic Device

Prior to establishing the microfluidic system as a model for micro capillary vessels, experiments were conducted in order to determine the most suitable materials, with respect to biocompatibility. The material should not have affected the proliferation and maintenance of the endothelial monolayer, but also should not have been degraded by the cells. Different materials were tested and the most suitable was chosen, based on the confluency levels after a cultivation time of 3–4 days.

The material selection was also influenced by the ease of the device assembly, since efficient seeding and assessment of cell confluency in the microfluidic system was simplified by fabricating a device that was made up of three different parts (see Figure 1).

The most suitable materials were cyclo-olefin polymer (COP) and cyclo-olefin co-polymer (COC), with 100% confluency of endothelial cells and PDMS with a cell confluency of 70% after three days. COP and COC are quite expensive materials and because of their structure, they would need to be thick in the cross-section, in order to be able to cut the microfluidic channels into the surface. This increased material thickness would, however, restrain the transparency of the device and thus restrict the live microscopic observation in the channels. Additionally, a device that was made from these materials would need to have additional seals between the different parts so as to avoid leakage after assembly. PDMS was chosen because it was less expensive, easier to manufacture, and there was no need for additional sealing of the parts. Furthermore, after the surface activation of the PDMS oxygen plasma treatment, a confluency rate of 100% was achieved in a time frame that was comparable to that using COC or COP, making PDMS the most suitable material for the microfluidic system, with regards to all of the design requirements.

The microfluidic device was comprised of three different parts. Two parts were made from PDMS, which contained the microfluidic channels. The upper channel, which represented the vessel equivalent, had a size of 500 µm × 100 µm × 5.9 cm (width × height × length). This channel was bordered on the lower side by a porous membrane with a pore size of 5 µm. The channel surface and the neighboring membrane were seeded with endothelial cells. The lower channel functioned as a cell trap and had a size of 1 mm × 1 mm × 5.9 cm (width × height × length). Immediately before the experiment, the three parts were mounted into an aluminum frame and infused with endothelial cell medium or the tumor cell suspension. As a result of the PDMS material properties, the additional bonding of the different parts was not necessary. The seeding of the single parts of the microfluidic device in an open setting before assembly was chosen for better control of homogeneous population and confluency of the endothelial cells. A tight confluent monolayer of endothelial cells, which was achieved after 24 h of cultivation in an open setting, were required before the assembly and the start of any experiment, in both the microfluidic channel, as well as on the adjacent membrane. This was essential to assure a tight barrier function of the endothelium along the entire dimension of the microfluidic channel for the extravasation experiments.

#### Coating of the Microfluidic Channel and Membrane

**Figure 3:**
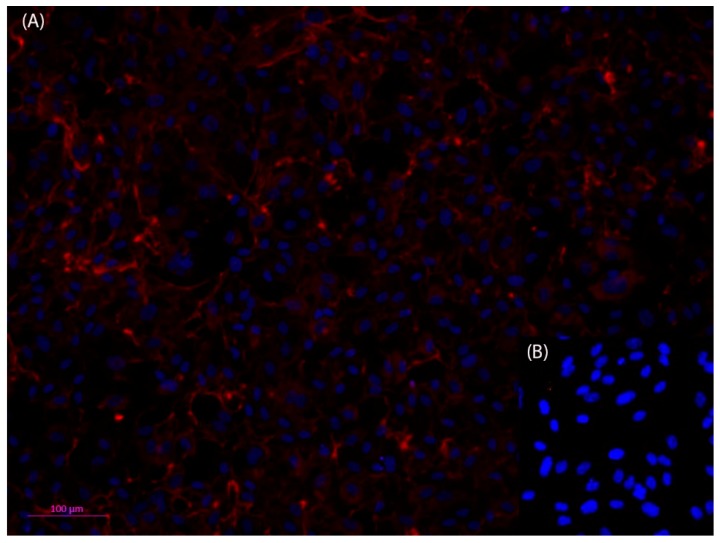
Fluorescent microscopic photos of anti-Collagen IV, Cy3 (red) immunofluorescence staining of human primary pulmonary arterial endothelial cells (HPAEC) (**A**); nuclei stained with Hoechst dye (blue). Collagen IV can be detected both in the cytosol of the cells as well as outside of the cells, which indicates that the HPAEC cells have formed an adequate basement membrane (**B**). The negative control shows that the method has been used appropriately. Dilution of antibodies: 1st antibody anti collagen IV 1:50, and 2nd antibody Cy3 anti rabbit 1:500). Scale bar 100 µm.

In the circulatory system, the inner vessel wall was arranged as monolayer endothelial lining, which rested on a basement membrane was composed of different extracellular matrix proteins, mainly collagen IV [43,44]. To better represent the vessel wall in the microfluidic device, the vessel equivalent was coated with different extracellular matrix proteins, like collagen I and laminin. However, within 24 h, the coating was disintegrated by the HPAEC endothelial cells that were used in this study (Figure A1). An anti-collagen IV immune fluorescence staining of the endothelial monolayer cultures in the open device revealed that the endothelial cells secreted collagen IV, and thus established their own basement membrane to rest on, which eliminated the need for coating with a supporting matrix (Figure 3).

### 3.2. Stability of the Endothelial Cell Monolayer within the Microfluidic Device

#### 3.2.1. Treatment of the Endothelial Cells with Flow

**Figure 4:**
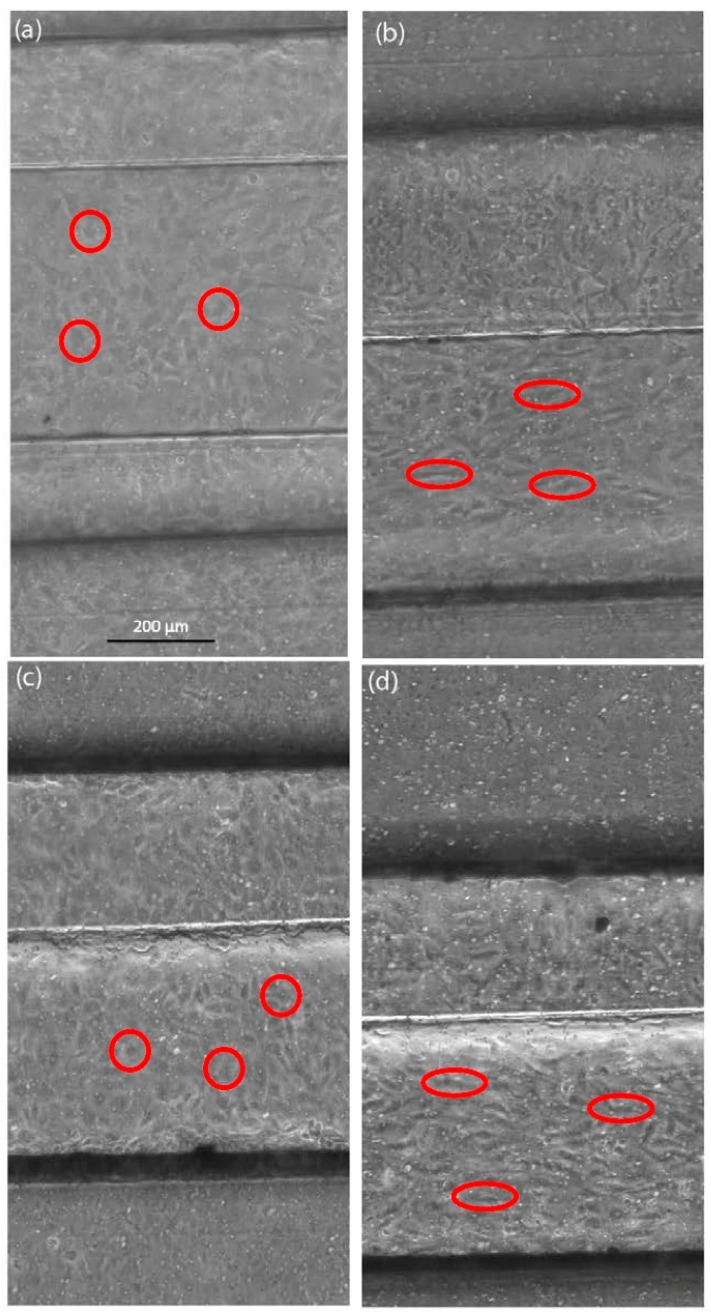
Live cell images of endothelial cells in the microfluidic device, perfusion flow velocity 24 mm/s. (**a**) Image of endothelial cells at the start of the continuous flow experiment; (**b**) Endothelial cells after continuous flow over 48 h; (**c**) Image of endothelial cells at the start of the pulse flow experiment; (**d**) Endothelial cells after the pulse flow perfusion at a rate of 60/min over 72 h. Before the flow is introduced, endothelial cells have a roundish shape (red circles). Endothelial cells show morphological changes, like elongation and orientation, into the direction of flow (red ovals), which might be influenced by the shear stress of the continuous or pulsating flow.

The functionality of the endothelial cell lining in the microfluidic channel was confirmed by perfusing the vessel equivalent channel with endothelial cell medium for up to 72 h, which used flow rates of 0.8 or 1.2 µL/s. The perfusion was either continuous or with a pulsation rate of 60/min. Figure 4 shows the endothelial lining within the microfluidic vessel equivalent, before and after perfusion.

Endothelial cells showed an elongated shape and an organization of the cell distribution in the flow direction. These morphological changes took place during the continuous as well as pulsation flow of 60/min, independent of the flow rate that was used. However, the changes took a longer time to become visible at lower flow rates. For the following experiments, the endothelial cell lining was treated with a continuous flow before the introduction of any tumor cells into the system.

#### 3.2.2. Immunofluorescence of VE-Cadherin

**Figure 5:**
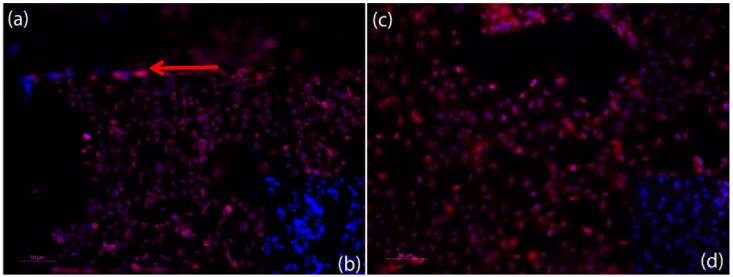
Fluorescent microscopic photos of anti-VE-Cadherin, Cy3 (red) immunofluorescence staining of HPAEC endothelial cells in the microfluidic device after finishing an experiment (nuclei stained with Hoechst) (blue). (**a**) Anti-VE-Cadherin Staining of the HPAEC endothelial cells within the microfluidic channel after an experiment. Red arrow shows edge of channel. VE-Cadherin can be detected on the surface of all endothelial cells and shows adherence junction expression; (**c**) Anti-VE-Cadherin staining of the HPAEC endothelial cells on top of the porous membrane of the microfluidic device after an experiment. VE-Cadherin can be detected on the surface of all of the endothelial cells and shows adherence junction expression. (**b**,**d**) Negative control for (**a**,**c**) show that method was conducted appropriately. Dilution of antibodies: 1st antibody anti VE-Cadherin 1:200, and 2nd antibody Cy3 anti rabbit 1:500). Scale bar: 50 µm.

The VE-Cadherin was a transmembrane protein that was involved in the mechanical stability of endothelial tissue and that also played an important role in the signal transduction pathways [45,46,47]. VE-Cadherin was an adherence junction protein and was expressed all over the cell surface area. On the inside of the cell membrane, VE-cadherin was connected to the actin cytoskeleton via catenin [45]. The membrane associated localization of the VE-Cadherin by immunostaining was considered as an adequate marker for the regular assembly of endothelial cell junctions. In the system that was introduced here, a VE-Cadherin immuno fluorescence staining was performed, which revealed a protein localization in the cell membrane, as an antibody for VE-Catherin was used, which indicated that cell–cell contacts were regularly expressed and integrated into the endothelial cell membrane. Figure 5 shows images of the endothelial lining in the microfluidic device after an anti-VE-Cadherin immunofluorescence staining.

### 3.3. Introduction and Adhesion of the Tumor Cells on the Endothelial Lining

**Figure 6:**
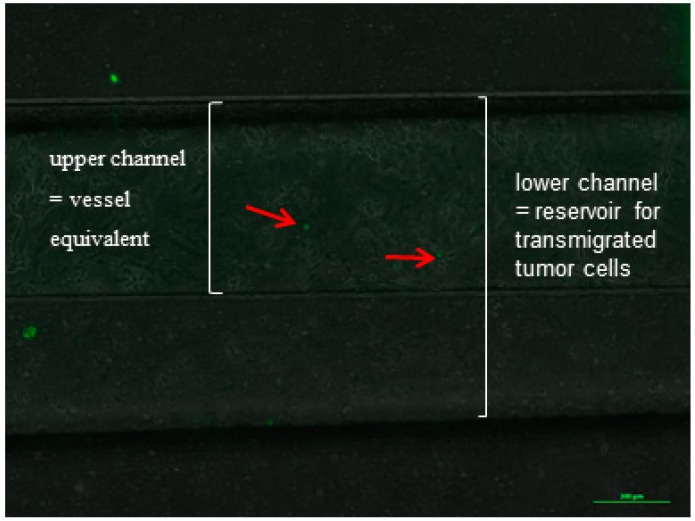
Example of an image of the microfluidic channel with endothelial lining (HPAEC) and adherent tumor cells (H838GFP) during an experiment, after a flow time of 60 min at a flow velocity of 24 mm/s. Endothelial cells were incubated with medium for 30 min before tumor cells were introduced into the microfluidic system.

The endothelial monolayer was incubated and perfused with medium for 30 min before the tumor cells were introduced to the system so as to ensure tight cell–cell contacts because of the exposure of flow in the microfluidic channel. The tumor cell suspension was infused for the duration of 6 h. After the end of the tumor cells circulation, the microfluidic system was disassembled and was fixed with 4% PFA for further analysis. The membranes and the microfluidic channels were microscopically analyzed and the adherent tumor cells that were easily identified because of the expression of GFP were counted (Figure 6, red arrows).

**Figure 7:**
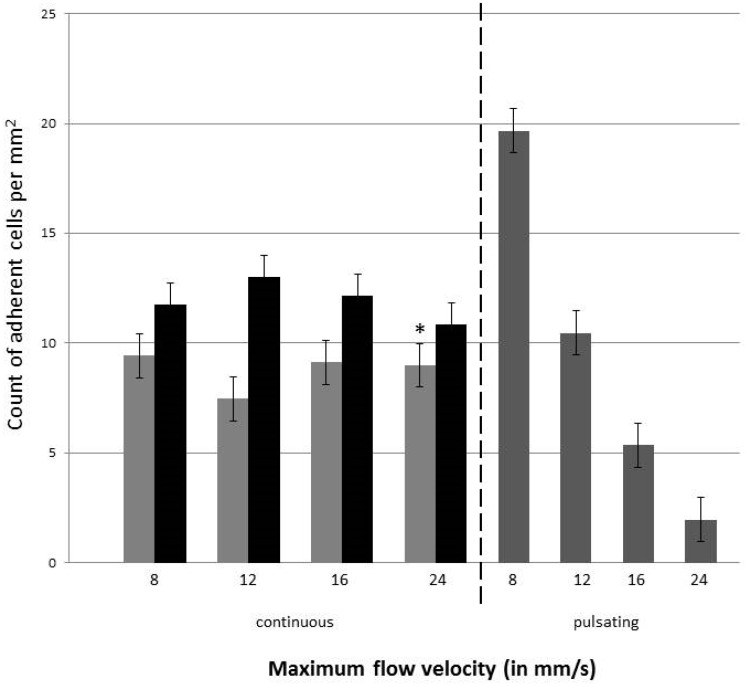
Number of H838GFP and SK-Mel 28GFP tumor cells adherent to the endothelial monolayer within the vessel equivalent. Light grey bars show the count for H838GFP, black bars the count of SK-Mel 298GFP for continuous flow, dark grey bars represent the number of H838GFP cells adherent to the endothelial cells when perfused with pulsatile flow. Four different flow velocities of 8, 12, 16, and 24 mm/s were used in both the continuous and pulsatile flow. Pulsation rate was 60/min. Endothelial cells were incubated with a medium for 30 min before the tumor cells were introduced. Tumor cell suspension was infused for 6 h. The mean value represents the average number of adherent cells in five different areas of 2 mm length in the microfluidic channel (top of the channel and membrane). The standard deviation is taken over the number of microfluidic devices used for the measurements. Dark grey bars and *: three devices were analyzed; light grey and black bars: 2 devices were analyzed.

Immediately before the experiment, the endothelial cells’ lined channel was perfused with a medium at a continuous flow velocity of 24 mm/s for 30 min. The tumor cells were introduced into the system with different flow rates, which ranged from 0.4 to 1.2 µL/s and corresponded to a flow velocity from 8 to 24 mm/s, respectively. The different experiments included a continuous and pulsatile flow. The tumor cells that adhered to the endothelial monolayer within the vessel equivalent were counted and the numbers were compared (Figure 7).

The data indicated that during the continuous flow, the number of adherent tumor cells in the endothelial cell-lined channel were not influenced by the flow rate, when they were perfused with a continuous flow. In contrast, when tumor cells were introduced into the system with a pulsating flow, more H838GFP cells adhered to the endothelial cells when the flow rate/velocity was lower. When comparing the number of adherent tumor cells at the same flow velocity, during either the continuous or pulsating flow, the number of cells was higher during the pulsating flow up to a flow velocity of 12 mm/s. At higher flow velocities, the number of adherent tumor cells were higher when they were perfused with a continuous flow.

In addition to the H838GFP lung carcinoma cells that were of epithelial origin, the microfluidic device was also perfused with SK-Mel 28GFP, which were cells of a mesenchymal origin, at a concentration of 5 × 10^5^ cells per mL. As for the H838GFP cells, the flow rate did not seem to influence the number of adherent cells to the endothelium under continuous flow. Interestingly, the comparison of the number of adherent H838GFP lung carcinoma and SK-Mel 28GFP melanoma cells to the endothelium, after an infusion time of 6 h, revealed a lower number of adherent lung carcinoma cells than of the adherent melanoma cells, independent of the flow rate that was used (Figure 7).

## 4. Discussion

For research on the metastatic cascade, it was crucial to develop 3D models of endothelial lined vascular systems, which could be perfused with either medium or tumor cells [14,23,26,31], and allowed an easy manipulation of the flow rates and shear stress in the vascular channel. Using this type of microfluidic system, the transendothelial migration, an essential step in successful metastasis of tumor cells, could be investigated under in vivo-like conditions [48]. To this end, a PDMS-based microfluidic system was introduced in this work. The device was assembled from three different parts, namely, two parallel channels with a porous membrane sandwiched in between. The membrane has a pore size of 5 µm and acted as the area for the transmigration of the tumor cells, while at the same time allowing a confluent population with endothelial cells, which mimicked the vessel wall. In the microfluidic devices that were introduced previously, the area for the transmigration was either defined by the microgaps of different sizes [49]; porous membranes with different pore sizes, ranging from 10 µm to 26 µm [31,36,38]; or the tumor cells and had to extravasate through the endothelial cells that were embedded in a hydrogel matrix [17,31,32,37]. These devices were limited in their comparability to the in vivo situation, since the establishment of a confluent endothelial cell layer on an authentic endothelial cell that was derived from ECM and a regular expression of endothelial cell junction proteins could not be guaranteed. 

In the device that was introduced here, the upper channel and the membrane were seeded with an endothelial cell monolayer in an open stage manner, prior to the assembly of the system. The concept of the open staged seeding and culture prior proved to be highly useful for achieving a confluent endothelial cell layer along the complete channel walls, which could easily be observed and controlled over the entire length of the microfluidic channel and the membrane that represented the vessel equivalent. The other microfluidic systems that were published were constructed with closed microfluidic channels, where seeding the of the endothelial cells took place by injecting the cells into the channel [17,30,36,37,38]. In these devices, the cells were incubated within the microfluidic channel from 10 min to 1 h, so as to ensure the adhesion of the cells to the coating of the microfluidic device [17,36,37]. Frequently, these studies failed to confirm a homogeneous population of the devices or to describe the difficulties in achieving a complete endothelial cell coverage. A control for the confluency of the endothelial monolayer was solely described by Jeon et al., using microscopic observation, before starting the extravasation experiments [17]. Cui et al. even described the technical difficulties so as to assure the confluent cell coverage over the whole membrane area [38]. These problems were resolved by the model that has been described here, since the control of a confluent endothelial monolayer throughout the entire device could be achieved microscopically before mounting the different parts to an experimental frame.

To ensure a high comparability to the in vivo situation, the endothelial cells that were used in this study were primary endothelial cells of the lung. The microfluidic vascular equivalents that were coated with these cells optimally represented the in vivo situation in metastasizing tumors—since the lung is one of the major homing sites for metastatic tumor cells of different origin. In contrast, the devices that were previously described by the other groups used human umbilical cord vein endothelial cells (HUVEC) [17,31,32] or human dermal microvascular endothelial cells (HDMVEC) that were isolated from the foreskin [31,36,38]. The seed and soil hypothesis, which was first published by Paget 1889, stated that the distribution of metastasis was not coincidental, but that different organs were ‘predisposed’ for the secondary tumor growth [19,50]. The establishment of a secondary tumor was thought to depend on the molecular communication of the tumor cells with the specific organ microenvironment, which included the endothelial cells that supported the survival and growth of the tumor cells [15,51]. Thus, because of the potential heterogeneity of the endothelial cells of the different tissues, the use of the primary endothelial cells, which were isolated from the metastatic site of interest, were to be preferred. Indeed, the studies showed significantly differed protein expression profiles in the HUVEC and endothelial cells of other origin, as well as the different behaviors of the foreskin endothelial cells versus the capillary endothelial cells from other organs [52]. While the HUVEC endothelial cells were frequently used in the model systems, the metastasis through the umbilical cord was a rare event with reliable data still lacking. Similarly, metastasis to the skin was very rare, with half of them being the outcome of the outgrowing tumor mass of the underlying primary tumor [53]. In contrast to these data, the metastasis to the lung was commonly seen in a number of tumors, such as breast, colorectal, kidney, head/neck, testicular and bone carcinomas, sarcomas, melanomas, and thyroid cancer [54,55]. Therefore, the HPAEC endothelial cells were chosen for the population of the microfluidic device in order to allow a good representation of the metastatic environment in vivo.

To mimic the blood vessel structure in vivo, it was important that the endothelial cell monolayer adhered to a basement membrane that was made up of extracellular matrix proteins, such as collagen IV and laminin, in the microfluidic vessel equivalent [56]. As a consequence of the microfluidic systems, which were established for the study of the metastatic cascade and were used for coating of the devices, mostly for better adhesion properties. The extracellular matrix proteins were most frequently used for coating either a collagen I hydrogel, matrigel, or poly-D-lysine [17,25,26,30,31,34,35,36,38]. While both collagen I and matrigel were not regular components of the vascular basement membrane [43], their use for coating was very common. To adapt the system that was presented here to a basement membrane, like the surface underneath the endothelial cells that coated the vessel equivalent microfluidic channel and porous membrane with ECM proteins, was tested. However, since the coating did not reliably adhere to the material and the endothelial cells disintegrated the extracellular matrix coating within 24 h after seeding, this approach was abandoned. The endothelial cells had been known to secrete a matrix metalloproteases to disintegrate and rebuild the extracellular matrix during angiogenesis and vascular remodeling [44]. The results that were obtained here suggested that the establishment of a confluent monolayer of endothelial cells in the channel was associated with the mechanisms that were seen during the vascular remodeling, which ultimately led to the disintegration of the extracellular matrix coating. In vivo, the endothelial cells were known to secrete components of the basement membrane [31], which mostly contained collagen IV fibers [25,45,46]. To determine whether the endothelial cells in the microfluidic device were able to establish their own basement membrane, an anti-collagen IV immune-fluorescence staining was performed on the HPAEC cells that were seeded in the vessel that was equivalent of the microfluidic device that was used in this work. The staining showed a partial staining for collagen IV in the cytosol of the endothelial cells, but also verified the secretion of collagen IV to the growth surface of the microfluidic system. Thus, it could be assumed that the endothelial cells that were used in this study were capable of establishing their own basement membrane, which made an additional coating of the device with the extracellular matrix proteins unnecessary. Thus, the system provided an authentic ECM for the attachment of the endothelial cells, which should have been better able to represent the in vivo environment. Specifically, collagen I and matrigel, which were frequently used in the microfluidic systems, were not regular components of the vascular basement membrane and could show the batch to batch variability and could therefore be a source of variable experimental results [43,47].

This was further confirmed by a regular expression of the cell junction protein VE-Cadherin at the cell surface of the endothelial cells that lined the microfluidic channel. The regular expression of the cell junction proteins, among them VE-Cadherin, was an essential characteristic of an intact endothelial cell lining in the vasculature [40]. While some publications failed to test the integrity of the endothelial cell monolayer in the system before the tumor cells were introduced [37], the analysis of the VE-Cadherin expression was a well-established method for microfluidic devices that used endothelial cells in order to examine the integrity of the endothelial cell monolayer. The regular staining against the VE-Cadherin all over the cell surface, without any gaps, which suggested a proper expression of cell–cell contacts in the system that was described here, was in agreement with the results from other research groups that characterized the endothelial cell lining of the microfluidic devices [17,34]. As such, the system provided an excellent platform for a dynamic capillary model.

In the new dynamic device that was introduced here, the seeded endothelial cells in the vessel equivalent could be exposed to flow, similarly to the in vivo situation. As a result, the endothelial cells showed morphological changes from a polygonal appearance to a more ellipsoid one, under high flow velocities. Additionally, they exhibited an orientation along the direction of the flow. Previous studies described similar changes upon the exposure of endothelial cells to the flow velocities and showed that the changes in the phenotype happened earlier than the changes in the cell alignment [57,58]. These variations and morphological changes in the appearance of the endothelial cells could have also been observed in the device that was used in this work. When adding a medium flow to the endothelial cell monolayer, within the vessel equivalent for more than 24 h, the changes of the phenotype and alignment occurred later during the pulsatile than during continuous flow, an observation that agreed with the study of Adams and Shaw [59].

In the microfluidic system that was established in this work, the tumor cells that were introduced to the endothelial cell-lined vessel equivalent were transfected with GFP, which enabled the identification of the tumor cells during the system perfusion via the live cell imaging, using a fluorescence microscope. The introduction of tumor cells into the system occurred at a flow rate, ranging from 0.4 to 1.2 µL/s, in either the continuous or pulsatile mode. These flow rates corresponded to the flow velocities of 8 to 24 mm/s. In the human aorta, the mean flow velocity was around 11 cm/s, however this vessel had a diameter of about 3 cm [60]. The flow velocities that were used for the microfluidic device that has been presented here, were much lower, yet the microfluidic channel, which acted as a vessel equivalent, only had the dimensions of 500 µm in width and was 100 µm high. In contrast, a human capillary vessel only has a diameter of 40–100 µm and a flow velocity of around 0.3 mm/s [60]. While the capillary flow velocity was significantly lower than the flow velocity that was used in the experiments that have been reported here, it is noted that the microfluidic channel had much larger dimensions, thus reducing the shear stress in comparison to a capillary at the same flow velocity. Other studies reported flow rates of 88 µL/min in a microfluidic channel with 2 mm width and 75 µm height [33]. This corresponded to a flow velocity of 10 mm/s, which was near the minimum velocity that had been tested with the microfluidic device that was presented here. Thus, the system that has been presented allowed for the application of a considerable shear stress and thus provided the basis for a systematic analysis of the influence of the shear stress and flow velocity on the tumor cell attachment to the endothelial cell lined vessel wall.

During the perfusion of the tumor cells through the microfluidic channel, the rolling as well as tight adhesion of these cells to the endothelial cells could be observed. The rolling process was characterized as loose adhesions that were broken off by the dynamic flow [7,61,62] and were considered a prerequisite for the tight adhesion and potential subsequent transendothelial migration of the tumor cells. While the flow velocity seemed to influence the tumor cell adhesion during the pulsatile flow, at a continuous flow—which was likely to be found in microcapillaries in vivo—the number of adherent tumor cells to the endothelium did not seem to depend on the flow velocity through the microfluidic system. Similar results were described by others [7,63]. In the work of Cui et al., the tumor cells were seeded on top of the endothelial cells and perfused afterwards with a rate of 20 µL/min of medium [39]. The transendothelial migration could be observed within 15 h, without flow [17], or after 24 h when the flow was applied to the microfluidic device [38]. In the experiments that were presented here, the rolling and the tight adhesion could be observed after 6 h. A transendothelial migration could not be observed within this time range, which make the cell trap excessive up to this time. In future tests, the infusion of the tumor cell suspension should have been extended to at least 24 h, so as to monitor the transendothelial migration processes.

## 5. Conclusions

In this study, a new microfluidic device is introduced in order to improve the issues occurring in the devices that have already published, particularly the control of the total confluency along the microfluidic unit, variabilities of cell behavior because of the extracellular matrix protein coating, and the use of primary endothelial cells from the metastatic target organs, which are more suitable for the research on metastatic processes. The devices that are used for research on tumor metastasis include the systems where the multilayer endothelial cells are embedded into a hydrogel to build a tubule-like vascular network. In other devices, the endothelial cells are seeded in the monolayer on top of an extracellular matrix protein coating along a microfluidic channel and the tumor cells can be introduced under flow conditions. These devices are mostly difficult to control for the confluency of the endothelial lining before starting an experiment.

The microfluidic device that has been introduced here consists of three parts, namely, two microfluidic channels and a porous membrane sandwiched in between. The upper, smaller channel, and the membrane acts as vessel equivalent and is seeded with primary endothelial cells that are isolated from the lung artery. This cell type was chosen since the lung is a favored site for the metastasis for many cancer types. The lower channel acts as reservoir to collect the extravasated tumor cells. The parts for the vessel equivalent can be seeded separately, with the endothelial cells in a concentration that is high enough to ensure a confluent monolayer over the whole length of the microfluidic channel. Confluency is controlled before the assembly of the device and at the start of any experiment. An additional coating of the device is not necessary for the endothelial cells that are used, as they secrete their own matrix within 24 h. The endothelial cell monolayer integrity was investigated using an anti-VE-Cadherin immuno-fluorescence staining and showed tight cell–cell contacts between the single cells of the monolayer. Under the flow conditions, the endothelial cells exhibited in vivo-like behavior, including the elongation of the cells and change of orientation in the direction of the flow. The tumor cells that were used for the study were the cancer cells of both epithelial and mesenchymal origin. The GFP transfected lung carcinoma cells H838 and malignant melanoma cell line SK-Mel 28 were introduced to the device as single cell suspension, under different flow conditions. The maximum flow rate ranged from 0.4 to 1.2 µL/s, using either a continuous flow or pulsatile flow with a rate of 60/min. The results show that the cancer cells adhere tightly to the endothelium under these conditions. In continuous mode, the number of adherent cells does not seem to depend on flow rate. Transendothelial migration could not be observed, as the experiments were terminated 6 h after the tumor cell introduction.

In summary, our results suggest that the device that has been introduced here can be used for the research on tumor cell extravasation and the mechanism of rolling, adhesion, and transendothelial migration of metastatic cells. The studies of chemokines, like CXCL 12 and TNF-α, as homing factors or adhesion inhibitors influencing the extravasation process, can be done by adding them either to the tumor cell suspension or into the reservoir, so as to collect the transmigrated tumor cells.
